# Robot-Assisted Laparoscopic Living Donor Nephrectomy: The University of Florence Technique

**DOI:** 10.3389/fsurg.2020.588215

**Published:** 2021-01-15

**Authors:** Sergio Serni, Alessio Pecoraro, Francesco Sessa, Luca Gemma, Isabella Greco, Paolo Barzaghi, Antonio Andrea Grosso, Francesco Corti, Nicola Mormile, Pietro Spatafora, Simone Caroassai, Alessandro Berni, Mauro Gacci, Saverio Giancane, Agostino Tuccio, Arcangelo Sebastianelli, Vincenzo Li Marzi, Graziano Vignolini, Riccardo Campi

**Affiliations:** ^1^Unit of Urological Robotic Surgery and Renal Transplantation, Careggi Hospital, University of Florence, Florence, Italy; ^2^Department of Experimental and Clinical Medicine, University of Florence, Florence, Italy

**Keywords:** kidney transplantation, living donor nephrectomy, minimally invasive surgery, robotics, technique

## Abstract

**Objective:** To provide a step-by-step overview of the University of Florence technique for robotic living donor nephrectomy (LDN), focusing on its technical nuances and perioperative outcomes.

**Methods:** A dedicated robotic LDN program at our Institution was codified in 2012. Data from patients undergoing robotic LDN from 2012 to 2019 were prospectively collected. All robotic LDNs were performed by a highly experienced surgeon, using the da Vinci Si robotic platform in a three-arm configuration. In this report we provide a detailed overview of our surgical technique for robotic LDN. The main objective of the study was to evaluate the technical feasibility and safety of the technique, including perioperative surgical complications rate and mid-term functional outcomes.

**Results:** Overall, 36 patients undergoing robotic LDNs were included in the study. Of these, 28 (78%) were left LDNs. Median (IQR) donor pre-operative eGFR was 88 (75.6–90) ml/min/1.73 m^2^. In all cases, robotic LDN was completed without need of conversion. The median (IQR) overall operative time was 230 (195–258) min, while the median console time was 133 (IQR 117-166) min. The median (IQR) warm ischemia time was 175 (140–255) s. No intraoperative adverse events or 90-d major surgical complications were recorded. At a median (IQR) follow-up of 24 months (IQR 11-46), median (IQR) eGFR patients undergoing in living donor nephrectomy was 57.4 (47.9; 63.9) ml/min/1.73 m^2^.

**Conclusions:** In our experience, robotic LDN is technically feasible and safe. The use of robotic surgery for LDN may provide distinct advantages for surgeons while ensuring optimal donors' perioperative and functional outcomes.

## Introduction

Live kidney donors are healthy individuals who intentionally undergo major surgery to improve the well-being of another individual; as such, maximizing the donor safety during this procedure is of paramount importance.

Several surgical techniques have been described for living donor nephrectomy (LDN), including open, pure or hand-assisted laparoscopic, natural orifice transluminal endoscopic surgical (NOTES) and robotic approaches (1–4).

In this view, minimally invasive techniques are increasingly being performed worldwide with the aim to further limit the morbidity of surgery for the donors while ensuring optimal grafts for kidney transplantation (5). While laparoscopic living donor nephrectomy (LDN) has become a common procedure in most Transplant Centers and has been shown to be associated with shorter hospital stay, less pain, and faster recovery as compared to open surgery (6, 7), the use of robotic surgery in this setting might further improve its perioperative outcomes providing distinct benefits for both donors and surgeons (1). This is mainly due to the advantages of the robotic platform as compared to standard laparoscopy (improved ergonomics, Endowrist technology, magnification and 3D vision). Consequently, robotic LDN has been implemented and is being increasingly performed at referral Centers with expertise in both living donor kidney transplantation and robotic surgery (8–10), with excellent outcomes, comparable to those of pure laparoscopic LDN (1).

In this report we provide a step-by-step overview of the University of Florence technique for robotic LDN, focusing on its technical nuances and perioperative outcomes.

## Patients and Methods

### Development of a Codified Robotic Living Donor Nephrectomy Program

A preliminary living donor kidney transplant program was set at our institution in 2002.

Between 2002 and 2011, 17 open living donor transplantations were performed. Of these, 15 LDNs were performed with an open approach while two with a pure laparoscopic approach.

In 2012, a well-codified robotic surgical program for LDN was established; this was followed by standardization of the surgical technique and development of a well-defined surgical team.

The transition to robotic surgery for performance of LDN occurred taking advantage of the progressive experience gained by our team in the field of open kidney transplantation (>650 from 1991) and robotic urologic surgery (for the treatment of prostate, bladder and kidney cancer).

A dedicated surgical team for robotic LDN was defined, including a surgeon who was highly skilled in robotic surgery and kidney transplantation (S.S.), bedside assistants experienced in laparoscopic surgery (urologists in training were allowed to assist together with a senior consultant), as well as highly skilled anesthesiologists and operating room nurses and support staff. This team remained essentially unchanged over the years.

### Patients and Dataset

After Institutional Review Board approval, data from patients undergoing robotic LDN from 2012 to 2019 were prospectively collected in an a priori developed web-based dataset.

All robotic LDNs were performed by a highly experienced single surgeon (S.S.). Since 2016, both LDN and kidney transplantation were performed in a twin operating theater, specifically designed to reduce the cold ischemia time during living-donor kidney transplantation (11–15).

Since 2017, kidney transplantation from living donors was also routinely performed with a robotic technique, introduced at our Center (11, 13, 15, 16) following a standardized program developed in collaboration with the European Association of Urology (EAU) Robotic Urology Section (ERUS) group (12, 14, 17, 18). At the beginning of our experience, RAKT was reserved for living donor kidney transplantation; afterwards, it was extended to deceased donors kidney transplantation following a well-codified framework (11).

Pre-operative assessment of living donors, as well as post-operative management and follow-up after LDN, were performed according to the latest EAU and KDIGO Guidelines (6, 19).

Donor candidates were evaluated by a multidisciplinary team. Donors had to be at least 18 years old and underwent a comprehensive pre-operative evaluation, including physical examination, laboratory tests, and psychiatric assessment.

A computed tomography (CT) angiogram and a renal scan with Tc 99m -DTPA (technetium-labeled diethylenetriaminepentacetate, a renal tubular function tracer) were performed by Institution protocol to evaluate both the donor's renal vascular anatomy and the split renal function.

The choice between right and left robotic LDN was always taken after a careful assessment of the donor's vascular anatomy (i.e., presence of multiple vessels and/or renal anomalies), split renal function at pre-operative renal scan, and surgeon's preference. No contraindication was set a priori regarding the use of right-sided kidneys.

Estimated Glomerular Filtration rate (eGFR) was calculated with the Chronic Kidney Disease Epidemiology Collaboration formula (20).

### Robotic Living Donor Nephrectomy: The University of Florence Technique

Robotic LDN was performed with a transperitoneal access using the da Vinci Si robotic platform (Intuitive Surgical Inc., Sunnyvale, CA, USA) in a three-arm configuration according to established principles (9).

The step-by-step overview of our surgical technique for left and right robotic LDN is provided in the Supplementary Video.

#### Step 1: Patient Positioning and Port Placement

After general anesthesia, the patient was placed in the right 70° lateral decubitus, for left nephrectomy, or in left 70° lateral decubitus for the right kidney (1, 9).

Port placement was performed according to the principles employed for radical nephrectomy for oncological reasons. In case of right LDN, one additional trocar (5 mm) was placed for liver retraction.

**Figure 1:**
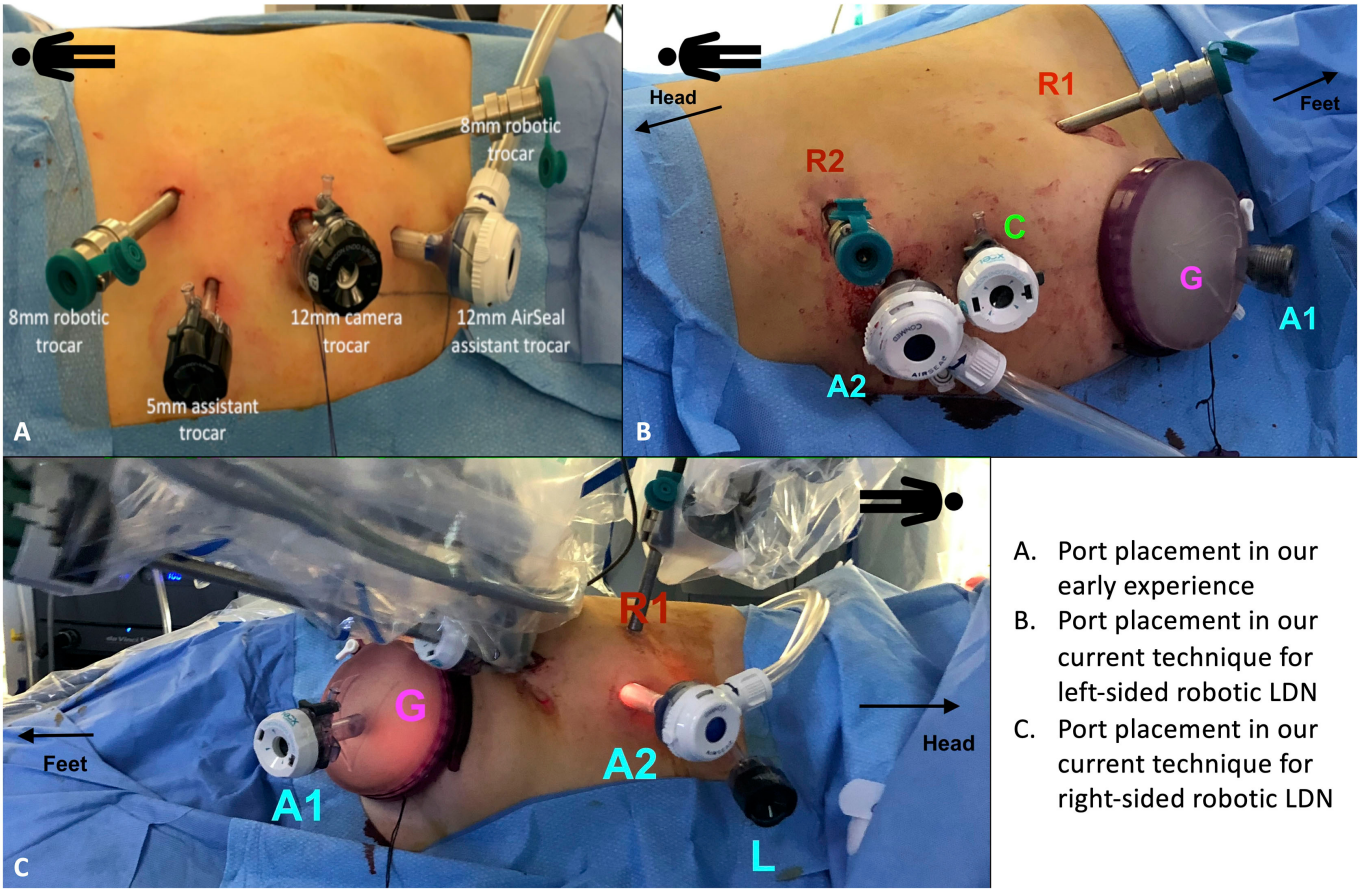
Port placement for robotic living donor nephrectomy (LDN). **(A)** Overview of the port placement for robotic LDN in the early phase of our experience. This included one 12 mm trocar for the camera, two 8 mm ports for the robotic arms and two assistant ports (one 12 mm and one 5 mm). The site for the Pfannenstiel incision was marked at the beginning of surgery but the incision (and the subsequent placement of the GelPOINT device) was made only after the kidney was entirely mobilized, just before controlling the renal vessels. **(B,C)** Overview of the current port placement for robotic left-sided **(B)** and right-sided **(C)** LDN (from 2017). The Pfannenstiel incision and placement of the GelPOINT device is performed at the beginning of surgery to save time and facilitate the kidney extraction. In this way, there is no need to re-dock the robot during the procedure. The AirSeal system is used routinely in all cases to maintain a constant pneumoperitoneum pressure of 10–12 mmHg.

Our technique involves the use of two robotic arms, a 12 mm trocar for the camera and two assistant ports (5 and 12 mm, respectively) (Figure 1).

Initially, the Pfannestiel incision used for kidney extraction was performed when the kidney was completely free from all surrounding tissues (i.e., just before ligation and section of the renal vessels) (Figure 1A). More recently, we opted for placing the GelPOINT^TM^ hand-assisted device immediately during port placement (at the beginning of surgery) in order to both achieve a quick access for kidney extraction and to switch to a hand-assisted laparoscopic procedure in case of emergency (Figures 1B,C).

The GelPOINT device is placed at the level of the Pfannenstiel incision, with a 12 mm laparoscopic assistant port placed within the Gelseal cap. This port is used to maintain the pneumoperitoneum pressure using the AirSeal device.

After section of the renal vessels, just before kidney extraction, a 15 mm endobag is introduced inside the abdomen through the GelPOINT device by creating a hole in the Gelseal cap while maintaining the 12 mm port in place for the assistant.

#### Step 2: Identification of the Retroperitoneal Anatomic Landmarks of Dissection

The procedure began with the medialization of the colon along the line of Toldt. Then, the classical retroperitoneal landmarks were identified (psoas muscle, ureter, and gonadal vein).

**Figure 2:**
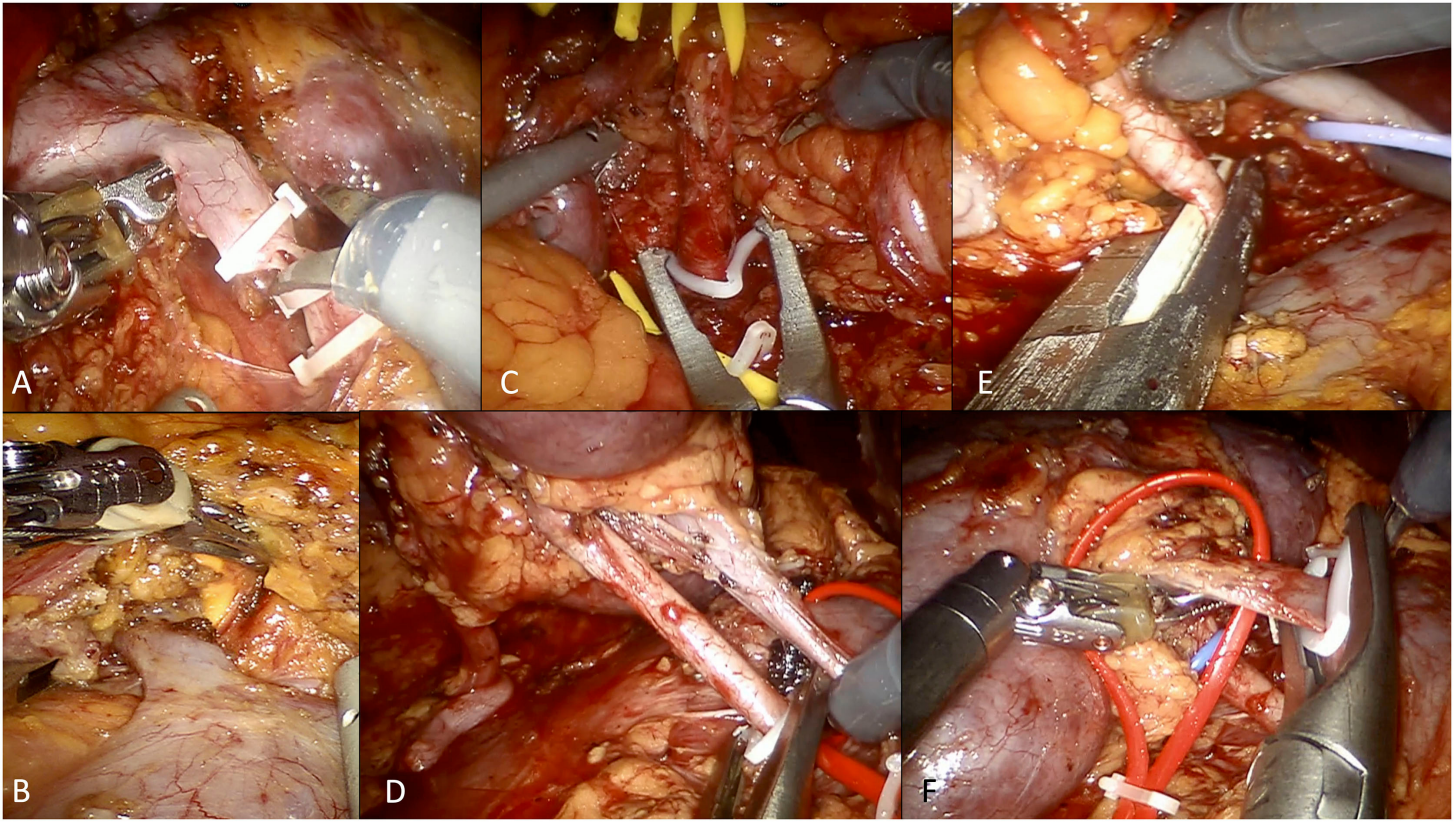
Overview of selected steps of robotic living donor nephrectomy according to the University of Florence technique. **(A)** Dissection and control of the left gonadal vein. **(B)** Isolation and dissection of the left adrenal vein. **(C)** Control of the left renal artery using Hem-o-Lok clips (at the beginning of our experience). **(D)** Control of the right renal artery using Hem-o-Lok clips (at the beginning of our experience). **(E)** Control of the right renal artery using a EndoGIA vascular stapler (current technique). **(F)** Control of the right renal vein using Hem-o-Lok clips (for the right renal vein, we currently prefer using the EndoGIA vascular stapler).

On both sides, after the medialization of the gonadal vein, the ureter was lateralized and freed from all surrounding tissues. Following the gonadal vein, the renal hilum was identified (Figure 2A).

#### Step 3: Isolation and Dissection of the Renal Hilum and the Kidney

Once the renal hilum was identified, the renal vein and artery (and all potential accessory vessels) were carefully isolated. The ascending lumbar vein was routinely identified on the left side, isolated and transected.

On the left side, the adrenal vein was always carefully dissected and ligated with two Hem-o-Lok clips (Figure 2B). Any additional gonadal or lumbar vessels were also controlled in order to free the entire length of the renal vein.

On the right side, the renal vein is shorter but there is usually no need to control the adrenal, gonadal, and lumbar veins as they drain directly into the inferior cava vein.

After hilar dissection, the kidney was entirely mobilized and isolated from the perirenal fat tissue.

During this step, a key point is the preservation of the so-called “golden triangle,” namely the space between the ureter, the lower pole of the kidney and the renal vein. Preserving this space is indeed thought to ensure an adequate vascularization of the ureter, potentially reducing the risk of ureteral ischemia.

#### Step 4: Management of Renal Vessels

Once the whole kidney was isolated, two Hem-o-lok clips were placed at the level of the distal ureter (at the level of its crossing with iliac vessels), which is then sectioned.

Initially, the renal artery was controlled using two Hem-o-lok clips, as previously described (Figures 2C,D) (21). Thereafter, from 2017 onwards, we used the EndoGIA vascular stapler, in line with the FDA warning contraindicating the use of Hem-o-lok clips during LDN (21) (Figure 2E).

The renal vein, as well as accessory arterial or venous vessels, were ligated using either the EndoGIA vascular stapler or two Hem-o-Lok clips, according to the caliber of the vessel and/or surgeon's preference (Figure 2F).

After section of the ureter and the renal vessels, the assistant surgeon introduced the 15 mm endobag through the Gelseal cap and easily removes the kidney together with the cap keeping warm ischemia time to a minimum.

Hemostasis is then achieved, and a drain is placed in the renal fossa. The kidney is then prepared for transplantation at the back table.

### Post-operative Management

Our standard enhanced recovery after surgery (ERAS) pathway for LDN included (a) early patient monitoring in a dedicated “recovery room” by the anesthesiologic staff for 4–6 h, with early removal of the nasogastric tube (at the end of surgery); (b) use of low-molecular-weight heparin subcutaneously (enoxaparin 4000 U), started on post-operative day 1, to prevent deep vein thrombosis; (c) removal of the surgical drain on post-operative day (POD) 1; (d) early patient mobilization (i.e., patients were placed out of bed for 2–3 h the day of surgery, had a ward ambulation at least twice a day on POD 1 and 2 until complete physical rehabilitation); (e) management of post-operative pain with intravenous drugs (i.e., paracetamol 1.0 g three times a day, ketorolac 30.0 mg IV two times a day) for 24–48 h after surgery, then oral analgesic therapy as clinically indicated.

### Study Objectives

The main objective of the study was to evaluate (a) the technical feasibility of robotic LDN, defined as completion of the operation without the need for open conversion and/or major intraoperative complications; (b) the safety of the technique, including perioperative surgical complications rate; and (c) the functional outcomes at a mid-term follow-up.

### Statistical Analysis

Descriptive statistics were obtained reporting medians (interquartile ranges [IQR]) for continuous variables, and frequencies and proportions for categorical variables, as appropriate.

Statistical analyses were performed using the Statistical Package for the Social Sciences (SPSS), version 24 (SPSS Inc., IBM Corp., Armonk, NY, USA).

## Results

Overall, 36 robotic LDNs were performed at our Center by a single surgeon during the study period.

### Donor and Graft Characteristics

The pre-operative clinical characteristics of the donors, including Charlson Comorbidity Index (CCI) (22), ASA score (23), are shown in Table 1.

**Table 1:** Pre-operative clinical characteristics of the donors included in our series.

**Pre-operative features (*n =* 36)**	
Age (years) (median, IQR)	55 (47–61)
Sex (number, %)	
• M	16 (44.5)
• F	20 (55.5)
BMI (kg/m^2^) (median, IQR)	25.6 (23.1–28.5)
Charlson comorbidity index (median, IQR)	1 (0–2)
Pre-operative creatinine (mg/mL) (median, IQR)	0.8 (0.7–0.95)
Pre-operative eGFR (ml/min/1.73 m^2^) (median, IQR)	88 (75.6–90)
Pre-operative Hb (g/dl) (median, IQR)	14 (13.4–14.9)
Previous abdominal surgery (number, %)	19 (53)
Previous malignancy (number, %)	
*Prostate cancer*	1 (3)
Race (number, %)	
• Caucasian	30 (83)
• Hispanic	3 (8)
• Asian	1 (3)
• Other	2 (6)
Smoking status (number, %)	
• Never	27 (75)
• Current smoker	2 (5)
• Former	7 (20)

Overall, the median (IQR) age was 55 (47–61) years and median (IQR) BMI was 25.6 (23.1–28.5) kg/m^2^.

One patient had a history of prostate cancer treated at our center 8 years before living donor nephrectomy with robotic-assisted laparoscopic prostatectomy (RALP). Oncologic follow-up was negative (PSA < 0.01).

Median (IQR) pre-operative creatinine was 0.8 (0.7–0.95) mg/dL and median (IQR) pre-operative eGFR was 88 (75.6–90) ml/min/1.73 m^2^.

Overall, 4/36 (11%) patients had a graft with two arteries and 1/36 (3%) had a kidney with two veins. For kidney transplantation, these cases were handled as follows: in one case, robotic kidney transplantation was performed with two separated arterial anastomoses to the external iliac artery; in the remaining three cases of multiple graft arteries, a single anastomosis was performed to the external iliac artery during robotic kidney transplantation after extracorporeal *ex-vivo* vascular reconstruction of the two arteries using a “pantaloon” technique; finally, in a case of a right-sided graft with two renal veins we performed a separated venous anastomosis to the external iliac vein during robotic kidney transplantation.

### Intraoperative Outcomes

Perioperative outcomes after the procedure are shown in Table 2.

**Table 2:** Intraoperative, post-operative and functional outcomes after robotic living donor nephrectomy in our series.

**INTRAOPERATIVE OUTCOMES**	
Overall operating room time (min) (from patient entry to exit the operating theater) (median, IQR)	230 (195–258)
Consolle time (min) (median, IQR)	132 (117–166)
Need to convert to open surgery during the robotic phases of the procedure (*n*, %)	0 (0)
Intraoperative complications (*n*, %)	0 (0)
Warm ischemia time (s) (median, IQR)	175 (140–255)
Cold ischemia time (min) (median, IQR)	75 (45–103)
**POST-OPERATIVE OUTCOMES**	
Length of hospitalization (days) (median, IQR)	6 (5–7)
ΔHb levels (g/dl) (POD 1 minus pre-operative) (median, IQR)	−1.3 (−0.3; −1.9)
ΔeGFR (ml/min/1.73 m^2^) (POD 1 minus pre-operative) (median, IQR)	−28.4 (−14.7; −37.1)
Creatinine at 1 month after surgery (mg/dL) (median, IQR)	1.2 (1.04; 1.4)
eGFR at 1 month after surgery (ml/min/1.73 m^2^) (median, IQR)	61 (54; 71.5)
Follow-up (months) (median, IQR)	24 (11; 46)
Creatinine at last follow-up (mg/dL) (median, IQR)	1.2 (1.03; 1.29)
eGFR at last follow-up (ml/min/1.73 m^2^) (median, IQR)	57.4 (47.9; 63.9)
ΔeGFR (ml/min/1.73 m^2^) (Last FU minus pre-operative) (median, IQR)	25.3 (16; 39)
Post-operative surgical complications (according to the modified Clavien-Dindo classification) (*n*, %)	9 (25)
• Grade 1 (*n*, %) (all <90d after surgery)	9 (25)
• Grade 2–5 (*n*, %)	0 (0)

*eGFR, estimated glomerulation filtration rate*.

In all cases, robotic LDN was completed without need of open conversion.

The median (IQR) overall operative time (from patient entry to exit from the operating theater) was 230 (195–258) min, while the median console time was 133 (IQR 117-166) min. The median (IQR) warm ischemia time was 175 (140–255) s.

Overall, 28/36 (78%) patients underwent left living donor nephrectomy.

No intraoperative adverse events were recorded in our series and no patients needed transfusions during or after the procedure. Overall, the median (IQR) length of hospitalization was 6 (5–7) days.

### Perioperative and Functional Outcomes

No major (Clavien-Dindo grade 3-5) (24) post-operative complications were recorded during the first 90 days after the procedure.

As shown in Table 2, median (IQR) decrease of hemoglobin and of eGFR were −1.3 (−0.3; −1.9) mg/dL and −28.4 (−14.7; −37.1) ml/min/1.73 m^2^, respectively, on post-operative day 1. No major late (>3 months) complications were recorded after surgery.

At a median (IQR) follow-up of 24 months (IQR 11-46), median (IQR) eGFR in patients undergoing living donor nephrectomy was 57.4 (47.9; 63.9) ml/min/1.73 m^2^. None of the donors was on dialysis or had an eGFR <30 ml/min/1.73m^2^ at last follow-up.

## Discussion

Improving technologies and integrating new strategies in surgical practice is a natural evolutive process, especially in challenging clinical scenarios such as kidney donation and transplantation.

In living kidney donation this process should aim to maximize donor safety, minimize donor discomfort (25, 26), and improve surgeon's ergonomics and confidence.

While pure laparoscopy has transformed LDN, making donation more appealing because of a reduction in LOS, pain, and convalescence, with a faster return to normal activity and improved cosmesis (21, 27), robotic surgery has the potential to further implement these benefits.

In this regard, the current EAU Guidelines on kidney transplantation consider laparoscopic LND as the preferred technique due to the robust evidence confirming its non-inferiority to open surgery regarding graft function, rejection rates, urological complications, and patient and graft survival (6).

Thanks to the 3D vision, high magnification and the Endowrist technology, as well as improved ergonomics, robotic LND might enhance the technical finesse of LND (1, 8). Moreover, robotic surgery might provide a framework for standardized training to potentially reduce the surgeon's learning curve for LND. Indeed, the number of transplant Centers that are performing robotic LDN is increasing (28), especially if performed by surgeons who are confident with robotic surgery.

Robotic LDN was performed for the first time in 2002 by Horgan et al. (29) who performed entirely robot-assisted laparoscopic LDNs on a cohort of 12 patients, showing the feasibility and safety of this procedure thanks to the benefits of robotic technology. These findings were confirmed by Hubert et al. (30) in 2007 on a series of 38 patients undergoing full robotic LDN. This trend is likely to increase in the near future. In addition, a recent randomized study showed that robotic LDN is safe and associated with a better morbidity profile than a pure laparoscopic procedure (31). Notably, the authors outlined that the robotic approach provides technical ease and facilitates preservation of longer length of renal artery on the right side.

However, it is important to highlight that current EAU Guidelines strongly recommend that robotic LND should be performed only in highly specialized Centers, in light of a large body of evidence stressing the importance of centralization of care to maximize post-operative outcomes (32, 33).

In this scenario, our experience confirms the benefits of robotic LDN from both a patient and surgeon point of view, providing key findings that may better contextualize the current literature on this topic.

First, robotic LDN was technically feasible, safe, and able to ensure optimal perioperative and mid-term functional outcomes. Of note, our experience is grounded on a high-volume of robotic urological procedures as well as on a large number of living donor kidney transplantation.

Conversion to open surgery was indeed not required in all cases, with no intraoperative adverse events or significant perioperative complications reported, in line with previous studies (34). Also, the median overall and console times required to complete robotic LDN in our series were similar to those reported by other groups (1, 34, 35).

A second key finding of our report is that, while being difficult to objectively evaluate with standardized metrics, the robotic approach offered a significant advantage to the surgeon concerning a reduction in intraoperative procedure-related stress. Moreover, the robotic platform facilitated the dissection of renal vessels in case of complex vascular anatomies (found in 5/36 patients)]. Of note, the surgeon could precisely manipulate the renal vessels (especially on the left side when the anatomy of the gonadal vein and of multiple accessory vessels is particularly challenging) and meticulously preserve a proper length of renal vessels, especially in case of right-sided kidneys (8/36 patients). Taken together, the benefits provided by the robotic technology in the manipulation of the right renal vessels might encourage surgeons to perform right robotic LDN in case of comparable baseline donor's split renal function (especially if the left kidney has vascular anomalies).

Finally, the use of the GelPOINT device, nowadays placed at the beginning of the procedure, allows the assistant surgeon to have a constant access to the operative field during all critical phases of the intervention, with the opportunity for an easy and rapid extraction of the kidney. Warm ischemia time in our experience was indeed comparable to that reported in laparoscopic series (34).

Thanks to the twin operating theater specifically designed for living donor kidney transplantation, also the cold ischemia time was relatively limited in our experience (Table 2).

As compared to the previously proposed techniques for robotic LDN, the potential benefits of our technique (from both patients' and surgeon's perspectives) are represented by: (a) the use of the GelPOINT hand-assisted device for rapid extraction of the graft after securing the renal hilum; (b) the early placement of the GelPOINT device at the beginning of the procedure, to facilitate the subsequent extraction of the graft and to avoid the need to temporarily stop the procedure to allow performance of the Pfannenstiel incision; (c) the placement of the GelPOINT device with a Pfannenstiel incision rather than a periumbilical incision, to improve cosmesis while ensuring optimal safety and time-efficiency; (d) the use of a two-row (rather than three-row) vascular stapling devices in case of short renal vessels (i.e., short renal vein during right-sided living donor nephrectomy or short accessory renal veins/arteries).

Notably, our experience confirms that robotic LDN can achieve optimal functional outcomes, as previously reported for laparoscopic LDN. In our series, at a median follow-up of 2 years, median (IQR) donor's eGFR was 57.4 (47.9–63.9) ml/min/1.73 m^2^.

Besides, at a median (IQR) follow-up time of 22.5 (9; 46.5) months, all donors were healthy, no late post-operative complications were recorded, and only five (14%) donors had an eGFR of <45 ml/min/1.73 m^2^.

Despite the increasing role of robotic surgery for LDN, it should be noted that the ultimate advantages of robotics over pure laparoscopy in the setting of LND are still matter of debate in both the Urology and Transplant communities (33, 36).

First, as compared to pure or a hand-assisted laparoscopic approaches, robotic LDN may be associated with higher operative time, ischemia time, costs, as well as with a “distance” between the operating surgeon and the patient (with subsequent need to rely on the bed-side assistant for potential emergency maneuvers). Moreover, robotic LDN may be more time-consuming as compared to a pure laparoscopic approach, given the need to dock the robot, undock it at the end of the procedure, as well as due to the “cost” of an inevitable learning phase. While also the time for kidney extraction might be theoretically an issue, the use of the GelPOINT device may significantly facilitate the kidney recovery and extraction, minimizing the warm ischemia time.

Regarding the higher costs of robotic surgery as compared to standard laparoscopy, it is important to note that the cost per-procedure at referral Centers could be paid off thanks to high volume of robotic surgeries performed yearly (1). In addition, the costs of robotic surgery are likely to be decreased in the future.

Concerning the length of hospitalization, our results may have been influenced by our early experience: in the first cases, we indeed tended to be more “conservative,” keeping patients in the Hospital for longer periods as compared to our current practice, which is based on enhanced-recovery after surgery (ERAS) protocols.

Of note, LDN is different from radical nephrectomy for kidney cancer, posing specific challenges to the surgeon. First, the procedure is conducted on healthy volunteers, so the safety of the donor is essential, and this inherently increases the surgeon's stress. Second, the ultimate aim is to provide the best quality of the graft to improve the recipient's outcomes, minimizing the possible intra- and post-operative complications. Finally, the dissection of the renal hilum can be challenging due to the presence of renal vascular anomalies and/or multiple vessels in a relatively frequent proportion of donors, making preservation of vascular length at times complex.

Overall, robotic technology might facilitate all these steps not only thanks to the characteristics of the robotic platform and instrumentation, but also thanks to the enhanced dexterity, ergonomics and subsequent surgeon's confidence, that overall allow for a more standardized and potentially easier procedure as compared to standard laparoscopy (36).

Finally, while there is currently lack of evidence on the impact of robotic surgery on the surgeon's learning curve for LDN, robotic technology may provide a standardized framework to develop a modular training model for this challenging procedure (33, 36–38), especially at academic Centers involving residents in kidney transplantation programs.

Our study is not devoid of limitations. All robotic LDNs were performed by a single surgeon with extensive experience in robotic urologic surgery, laparoscopic LDN and kidney transplantation, at a high-volume academic Center performing >1,000 robotic procedures/year. As such, our findings might not be generalizable in other clinical settings. Moreover, our experience includes a relatively small number of patients with a relatively short follow-up. Thus, larger studies with longer follow-up are needed to confirm our results and to assess the ultimate benefits of robotics for LDN.

In conclusion, robotic LDN is technically feasible and safe in experienced hands and in referral transplant Centers. While ensuring all benefits of laparoscopic surgery, the robotic technology has the potential to further improve the technical finesse of LDN and minimize the intraoperative surgeon's stress, especially in patients with complex vascular anatomy.

Given the current trend toward an increased use of robotic surgery for urological and transplant procedures worldwide, our preliminary experience represents another step forward in this direction, confirming optimal perioperative and mid-term functional outcomes of robotic LDN.

Further studies are needed to confirm our findings and to define the ultimate benefit of robotic surgery for LDN.

## Data Availability Statement

The data analyzed in this study is subject to the following licenses/restrictions: The dataset is available upon request. Requests to access these datasets should be directed to Riccardo Campi, riccardo.campi@gmail.com.

## Ethics Statement

The studies involving human participants were reviewed and approved by Ethical Committee of Careggi University Hospital (Comitato Etico Area Vasta Centro), Florence, Italy. The patients/participants provided their written informed consent to participate in this study.

## Author Contributions

RC, FS, GV, AP, and SS: study design. NM, PB, FC, AG, AB, AP, and LG: data collection. RC: statistical analysis. RC, AP, and FS: manuscript writing. MG, AS, VL, SG, PS, SC, AT, GV, and IG: critical revision of the manuscript. SS: supervision. All authors: contributed to the article and approved the submitted version.

## Conflict of Interest

The authors declare that the research was conducted in the absence of any commercial or financial relationships that could be construed as a potential conflict of interest.

## Supplementary Material

Supplementary Video Step-by-step University of Florence technique for robotic living donor nephrectomy.
